# Pathophysiological Impact of Cigarette Smoke Exposure on the Cerebrovascular System with a Focus on the Blood-brain Barrier: Expanding the Awareness of Smoking Toxicity in an Underappreciated Area

**DOI:** 10.3390/ijerph7124111

**Published:** 2010-11-26

**Authors:** Peter Mazzone, William Tierney, Mohammed Hossain, Vikram Puvenna, Damir Janigro, Luca Cucullo

**Affiliations:** 1 Department of Pulmonary Medicine, Cleveland Clinic Lerner College of Medicine, E 100^th^ St, Cleveland, OH 44106, USA; E-Mail: MAZZONP@ccf.org (P.M.); 2 Case Western Reserve University, 10900 Euclid Ave., Cleveland, OH 44106, USA; E-Mail: wst9@case.edu (W.T.); 3 Cerebrovascular Research, Cleveland Clinic, 9500 Euclid Ave., Cleveland, OH 44195 USA; E-Mails: Hossaim@ccf.org (M.H.); janigrd@ccf.org (D.J.); Puvennv@ccf.org (V.P.); 4 Department of Cell Biology, Cleveland Clinic, 9500 Euclid Ave., Cleveland, OH 44195, USA; 5 Department of Molecular Medicine, Cleveland Clinic Lerner College of Medicine, E 100^th^ St, Cleveland, OH 441065, USA

**Keywords:** tobacco smoke, alternative, blood-brain barrier, central nervous system, inflammation, endothelial cells, white blood cells

## Abstract

Recent evidence has indicated that active and passive cigarette smoking are associated, in a dose-dependent manner, with dysfunction of normal endothelial physiology. Tobacco smoke (TS) may predispose individuals to atherogenic and thrombotic problems, significantly increasing the risk for ischemic manifestations such as acute coronary syndrome and stroke. Despite the strong evidence for an association between smoking and vascular impairment, the impact of TS exposure on the blood-brain barrier (BBB) has only been marginally addressed. This is a major problem given that the BBB is crucial in the maintenance of brain homeostasis. Recent data have also shown that chronic smokers have a higher incidence of small vessel ischemic disease (SVID), a pathological condition characterized by leaky brain microvessels and loss of BBB integrity. In the brain TS increases the risk of silent cerebral infarction (SCI) and stroke owing to the pro-coagulant and atherogenic effects of smoking. In this article we provide a detailed review and analysis of current knowledge of the pathophysiology of tobacco smoke toxicity at the cerebrovascular levels. We also discuss the potential toxicity of recently marketed “potential-reduced exposure products”.

## 1. Introduction

A great deal is known about the pathophysiologic and clinical effects of tobacco smoke exposure. Tobacco smoke exposure is responsible for a large portion of the preventable deaths worldwide. Knowledge of its impact on vascular health, particularly related to cardiac disease, is well established. Less well described are the mechanisms of tobacco smoke induced vascular damage in the cerebrovascular system, particularly as they apply to the blood brain barrier.

## 2. Tobacco Smoke Induces Oxidative Injury to the Cerebrovascular System

Oxidative stress is induced by reactive oxygen species (ROS) which are either free oxigen radicals or reactive anions containing oxygen atoms. These highly reactive species can then interact with molecules containing oxygen atoms and propagate the production of other free radicals. Accumulation of ROS is the result of one of these three factors: (1) an increase in oxidant generation, (2) a decrease in antioxidant protection (e.g., shortage of ascorbic acid, α-tocopherol, Coenzyme Q10 ) [1–4], or (3) a failure to repair oxidative damage. Under normal conditions, ROS are cleared by the intracellular action of superoxide dismutase (SOD), catalase, glutathione (GSH) peroxidase [5] or (extracellular) antioxidant vitamins such as ascorbic acid (vitamin C), and α-tocopherol (vitamin E).

**Figure 1:**
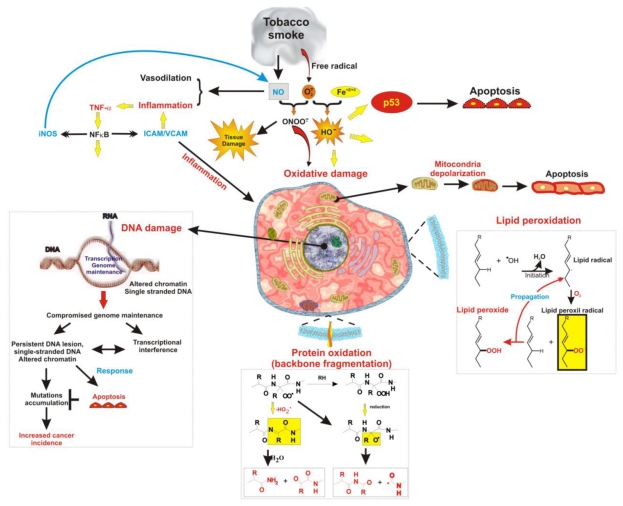
ROS-induced cellular inflammatory response and oxidative damage. Schematic representation of the multiple pathways by which the exposure to reactive oxygen species originated by tobacco combustion can induce cellular damage and inflammation.

Unfortunately tobacco smoke contains very high levels of superoxide and other reactive oxygen species (such as hydroxyl radical, hydrogen peroxide, and peroxynitrite) derived from cigarette combustion. Therefore, the vascular adverse effects of smoking can be the result of endothelial exposure to ROS (Figure 1) [6–11]. Damage to cells occurs as a result of ROS-induced alterations of macromolecules [12]. These includes lipoperoxidation of polyunsaturated fatty acids in membrane lipids, protein oxidation, DNA strand breakage [13–16], RNA oxidation [17], mitochondrial depolarization and apoptosis. Mutations of the nuclear protein p53 which may lead to apoptosis are also associated to tobacco smoke toxicity. Specifically to direct DNA damage from carcinogens contained in cigarette smoke [18–20]. *In vivo* and *in vitro* studies have shown that antioxidant supplementation prevents, to some extent, the oxidative damage and inflammation induced by cigarette smoke exposure [21–23], thus strongly supporting the hypothesis of a ROS-mediated toxicity of tobacco smoke exposure. Additionally, oxidative stress and ROS have been implicated in disease states, such as Alzheimer’s disease [24], Parkinson’s disease [25], various cancers [26], and aging processes [27].

## 3. Tobacco Smoke Induces Inflammatory and Thrombotic Injury

Components of cigarette smoke also contribute to a pro-atherosclerotic environment by triggering a complex pro-inflammatory response through the recruitment of leukocytes to the site of inflammation via cytokine signaling (such as IL-1β and TNF-α) [28], matrix metalloproteinase upregulation (e.g., MMP-1 and MMP-9), and by promoting the adhesion and binding of monocytes to the endothelial wall of blood vessels [29]. Inflammatory activation of endothelial cells (ECs) leads to an increased expression of selectins, VCAM-1, and intercellular adhesion molecule-1 (ICAM-1) [30]. This promotes the adherence of monocytes to vessel walls. Elevated levels of white blood cells, primarily neutrophils and monocytes are observed in smokers [31]. In particular, neutrophils secrete free radicals, elastase and collagenase [32] which are thought to contribute directly to EC injury as they add to the immune response.

Despite the fact that smoking triggers significant pro-inflammatory activity and active smokers therefore, have significantly higher number of circulating white blood cells [33], they are generally more susceptible to viral and bacterial inflammatory neuropathologies than non-smokers [34]. This suggests that chronic smoking causes desensitization rather than a potentiation of the response to other inflammatory stimuli and may undermine the ability of the host immune system to counteract viral and bacterial infection. Desensitization of the host immune response, in addition to the compromising effect on the BBB integrity, can facilitate the pathogenesis of neurological disorders such as bacterial and viral meningitis [35–38].

Smokers are also reported to be at increased risk for thrombosis. Platelet activation is frequently observed in smokers in response to increased levels of platelet activating factor, Von Willebrand factor, catecholamines, and thromboxane. This phenomenon has been confirmed *in vitro* [39] and *in vivo* [40]. All these factors pose a serious threat at the level of brain microvasculature where vascular tone regulatory mechanisms are absent. Elevated C-reactive protein (CRP) levels caused by cigarette smoking, can also promote endothelial dysfunction by lowering the production of nitric oxide (NO) and diminishing its bioactivity [41]. Recent studies demonstrated that CRP can decrease eNOS mRNA, augment ET-1, and upregulate nuclear factor κB (NF-κB) signaling in ECs while attenuating endothelial progenitor cell survival and differentiation.

## 4. Pathophysiology of BBB Endothelial Cells

The BBB has been shown to maintain brain homeostasis. It selectively excludes most endogenous and xenobiotic blood-borne substances from entering the brain, protecting it from systemic and exogenous influences [42–45]. The BBB dynamically responds to hemodynamic disturbances (e.g., focal ischemia), through free radical release and cytokine generation. It also plays a crucial role in protecting against neurotoxicity. Dysfunction of the BBB is involved in the pathogenesis and progression of a number of neurological disorders (including stroke, multiple sclerosis, Alzheimer’s disease, dementia, epilepsy, *etc.*) [46]. Any disorder affecting BBB function may have secondary effects on cerebral blood flow and vascular tone, further influencing transport across the microvascular endothelium. For example, cigarette smoke has been shown to lead to cerebrovascular vasodilation through sympathetic activation. Nicotine activates nicotine receptors, which leads to the acetylcoline-dependent release of NO from the vascular endothelium [47,48] through activation of endothelial nitric oxide synthase (eNOS) [49]. NO is one of the major endothelium-derived relaxing factors, which plays an active role in regulating microvascular tone and the cerebral blood flow under normal and pathological conditions [50]. Furthermore, NO has been shown to increase vascular permeability at the BBB thus impairing brain homeostasis and facilitating the passage of unwanted substances from the blood into the brain [49,51,52].

Trans-endothelial leukocyte migration across an altered BBB is one of the most prominent features of many neuroimmune disorders; leukocytes are found in large numbers in the brain following trauma and certain neurodegenerative diseases. It is not clear whether the cells cross the endothelium through tight junctions, via a large pore or vacuole in the EC, or through some other mechanism [53]. The passage of cells across the BBB occurs when several cell types (blood cells and endothelial and/or glia) are activated [54]. Vascular EC at the site of inflammation undergo a number of morphologic and functional alterations, including increased permeability, hypertrophy, the accumulation of intracellular organelles, and proliferation [55].

Exposure of endothelium to pro-inflammatory cytokines (TNF-α and IL-1β) interrupts the BBB by disorganizing cell-cell junctions, decreasing the brain solute barrier and enhancing leukocyte endothelial adhesion and migration. Despite the crucial importance of the BBB and the strong scientific and clinical evidence for an association between TS exposure and vascular impairment, the effects of smoking on the BBB have been only marginally addressed and studies have been limited to a handful of substances among the multitudes found in cigarette smoke.

## 5. Vascular and Inflammatory Effects of Tobacco Smoke on the BBB

**Figure 2:**
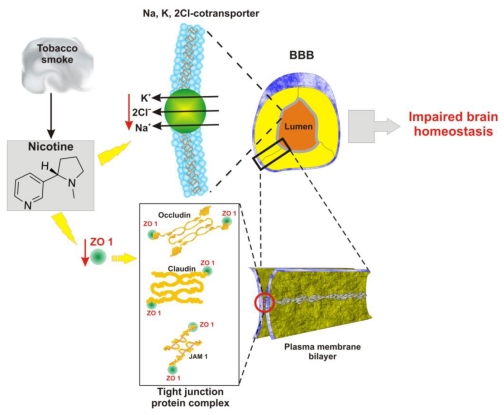
Exposure to nicotine impairs BBB function. Nicotine decreases expression of ZO1, which is a critical component of a variety of tight junctional proteins and that of the Na, K, 2C co-transporter. This can lead to impaired BBB function and altered brain homeostasis.

Tobacco smoke contains over 4 thousand chemicals (over 4 dozens of them are well established carcinogens). Direct and second hand exposure to tobacco smoke are associated with a number of physiological vascular changes that can lead to the pathogenesis of cerebrovascular-related diseases. For example, nicotine contained in tobacco smoke has been shown to negatively effect endothelial tight junctions [56] and the brain-to-blood Na^+^ K^+^ 2Cl^−^ co-transporter located on the luminal surface of BBB [57] (Figure 2).

Increased blood viscosity, related to TS, can lead to impairment of blood flow, risking the integrity of the brain microvasculature, particularly if the inter-endothelial tight junctions are already compromised. Increased levels of matrix-degrading and proinflammatory changes in vascular EC exposed to cigarette smoke have been found [29,58].

In addition to Nicotine, which has been proven to be potentially harmful to the integrity and function of the BBB [56,57,59–61], ROS promotes low-density lipoprotein oxidation which at the vascular level can cause cell injury and formation of atherosclerotic lesions [9,62]. At the BBB level ROS may induce pinocytosis, thus increasing transcytotic activity across the BBB endothelium [8] but can also cause direct BBB breakdown (especially in conditions like stroke and traumatic brain injury [63]). This occurs by tight junction (TJ) modification, local matrix metalloproteinases (MMPs) activation and basal membrane degradation [63]. ROS and nicotine act synergistically with other potentially harmful systemic stimuli (e.g., hypoperfusion of the brain vessels) to further impair both BBB function and integrity and leads to secondary brain damage. This hypothesis is supported by previous studies by Yin *et al.*, [64] and by our recently published data demonstrating that BBB impairment associated with a transient loss of flow is significantly worsened by TS exposure [58]. Furthermore, in BBB endothelial cells TS exposure induced a significant transcriptional upregulation of genes involved in the inflammatory response. Chemokines (CCL2, CXCL1, CCL5, *etc.*), pro-inflammatory cytokines (IL-8 IL-1β, *etc.*), STAT3, (which is an essential regulator of the anti-inflammatory function of ECs in systemic immunity [65,66]), and other genes involved in the modulation of the endothelial inflammatory response to TS where all significantly upregulated [58]. In summary smoking and hemodynamic impairments can synergistically contribute to vascular inflammation and BBB damage.

Cigarette smoke contains high concentrations of NO [67], which may affect the viability of the BBB. Nitric oxide is a critical factor that affects the vascular tone, modulates platelet aggregation and leukocyte adhesion to the endothelium. At the BBB, NO plays an inhibitory role in the dynamic regulation of BBB function [68,69] and is involved in a variety of physiologic and pathological processes as part of the inflammation process itself. Early during ischemic injury, NO has a vasodilatatory effect, mediated by *endothelial nitric oxide synthase* (eNOS), which seems to be protective for the brain [70]. This is followed by massive production of inducible NO (iNOS), which peaks at 12–48 h after ischemia and occurs in inflammatory cells infiltrating the brain and in the cerebral blood vessels. Through a process of redox cycling that diverts NO toward peroxynitrite formation nitric oxide is inactivated and used for the production of the superoxide anion radical O^2 −^ [7]. This is a highly reactive oxygen species that propagates inflammation to adjacent districts and extend the damage. In addition to NO systemically introduced by smoking, TS can also modulate the level NO by decreasing the activity of eNOS and promoting that of its inducible form (iNOS) [71]. The result is the initiation and progression of vasculo-pathogenic diseases such as atherosclerosis, thrombosis and ischemic like insults.

## 6. Consequences of TS Induced Damage

One mechanism by which smoking can harm health is directly related to hampered BBB viability and function. This facilitates the pathogenesis and progression of a number of neurological disorders [46,72,73]. There is indeed a correlation between smoking with an increased risk for multiple sclerosis [74], Alzheimer’s disease [75], and neurodevelopmental damage during pregnancy [76,77].

### Small vessel ischemic disease

Chronic smokers have a higher incidence of small vessel ischemic disease (SVID) than non-smokers [58]. SVID is a pathological condition characterized by leaky brain microvessels and loss of BBB integrity. As part of an independent study started over a year ago by Cucullo *et al.*, serum measurement of S100β, a marker of BBB integrity [72–74], has clearly shown a significantly higher degree of BBB disruption in smokers than non-smokers. This finding was further corroborated by Magnetic Resonance Imaging (MRI) scans showing widespread white and grey matter signals consistent with impaired BBB function [58], and by the presence of leaky microvessels. The study revealed that smokers have a significantly higher propensity (83% of the patients) for cerebrovascular changes that lead to gadolinium enhancement and/or positive flair signals in the brain than non-smokers (36% of the patients).

### Cerebrovascular injuries

Cigarette smoking increases stroke incidence and brain infarction by approximately 50% [75,76] with a risk that raises proportionally with the amount of exposure whether derived from direct [77] or second hand smoking [78]. The increased stroke risk caused by smoking has been attributed to both pro-coagulant and atherogenic effects [79,80]. In particular it has been shown that TS causes a dose dependent oxidant-mediated stress responses, cell death in vascular endothelial cells, and circulating monocytes which are the major cellular player in the induction of atherosclerotic lesions [81]. A substantial relationship has been established between TS exposure and the onset of silent cerebral infarction (SCI) that is comparable with that of known cerebrovascular risk factors such as hypertension [82]. SCI is a known cause for progressive brain damage resulting in vascular dementia.

A recent study by Hossain *et al.* suggests that an ischemic-like event is likely to induce a stronger inflammatory response in smokers than non-smokers [58]. Relevant to the secondary post-ischemic brain injuries (and therefore, to the pathogenesis of many neurological and neurodegenerative diseases) is the pro-inflammatory stimulus of TS (increased levels of IL-6, TNF-α, IL-1β and other pro-inflammatory cytokines) [83] to which the BBB dynamically responds. This facilitates the pathogenesis and progression of a number of neurological disorders [45,84,85].

### Behavioral impairment and increased risk for sudden infant death syndrome

The brain serotonin (5-HT) system has been demonstrated to play a major role in central nervous system (CNS) development, cognitive (memory and learning), and personality and behavioral modulatory processes. In fact, several neuropsychiatric conditions (e.g., obsessive compulsive disorder, anxiety, depression, schizophrenia, *etc.*) as well as impaired brain functions (e.g., sleep disorders, appetite, *etc.*) have been related to an altered serotonin (5-HT) system. Recent studies in pregnant Rhesus monkeys exposed to environmental tobacco smoke have clearly shown specific (5-HT) receptor deregulation in the developing neonates and suggest that this may be responsible for behavioral abnormalities associated with perinatal tobacco exposure [86]. Furthermore, recent studies have clearly shown a link between impaired BBB function with the onset of depression and schizophrenia [87].

According to studies by Teaktong *et al.*, acute inhibition of serotonin neurons, which control a wide range of behavioral and physiological processes, is primarily related to an effect on nicotine receptors [88,89]. Prenatal and early postnatal exposure to tobacco smoke has also been associated with an increased risk for sudden infant death syndrome (SIDS). Experiments performed in pregnant monkeys suggest that the effect of tobacco smoke exposure on SIDS may be mediated by respiratory problems associated to neuroplastic changes in the nucleus of the solitary tract (NTS) [90] where lung sensory information and respiratory function are first integrated. Furthermore, recent studies have shown an increased expression of active caspase-3 (a marker of cell apoptosis) in the brainstem of SIDS infants who have been exposed to passive smoke [91].

## 7. New Tobacco Products: Lower Risks or More of the Same?

Besides elevating the risk of at least nine forms of cancer [92], smoking is linked to heart disease, emphysema, and pulmonary disorders.

The issuance of the NCI’s Monograph 13 on “light” and “low tar” cigarettes demonstrates the problem of relying on tobacco industry claims of reduced risk for its products in the absence of meaningful government regulation and oversight. Light and low tar cigarettes were introduced with claims of a reduction in harmful components and implied health benefits. Decades later and after millions of health concerned consumers switched to these products, the evidence demonstrates that these expectations were false. Today the tobacco industry is introducing a whole new line of products with claims and representations that are stunningly similar to those made for light and low tar products when they were introduced. However, in the absence of government regulation of these products and claims, the American public has no greater certainty that the claims being made for these new products are any more reliable than the claims made for light and low tar cigarettes.

OMNI and Advance are two examples of so-called “reduced risk cigarettes” or “potential reduced exposure products” (PREPs) that have recently become available in the stores in the United States. Advance cigarettes are made with a special tobacco-curing process and a filter that reduce nitrosamines and hydrocarbons. Omni also uses tobacco processed to reduce nitrosamines and hydrocarbons. Both products have been marketed as less dangerous alternatives to smoking traditional cigarettes. Smokeless or non-combusted oral tobacco products are also gaining greater momentum and interest by both the public health community and the tobacco industry. These products (such as Revel manufactured by the U.S. Smokeless Tobacco Company; USSTC and Camel Snus manufactured by Swedish Match) were introduced as a “safe” replacement for cigarette smoking.

In both cases the claim of “decreased risk” is dubious because: (1) there is no proof that reducing hydrocarbons or nitrosamines translates to a decreased risk to smokers; (2) although the overall exposure to potentially harmful substances generated during cigarette combustion is lower with PREPs than with regular cigarettes, addiction to tobacco use is not reduced [93]; (3). Despite the fact that PREPs may not expose the user to some of the potentially noxious compounds associated with combustion, they still contain over 2 dozens carcinogens including N’-nitrosonornicotine (NNN) formed during the curing, processing, and aging of tobacco [94,95]; (4). With up to 4,000 chemicals present in cigarette smoke, the risks to smokers are varied and many still unidentified. Therefore it is impossible at the current stage to affirm that PREPs are a safe replacement for conventional tobacco products. This is a remarkable public health issue because smokers may be misled into assuming that smokeless and reduced-exposure tobacco products are actually safe.

**Figure 3:**
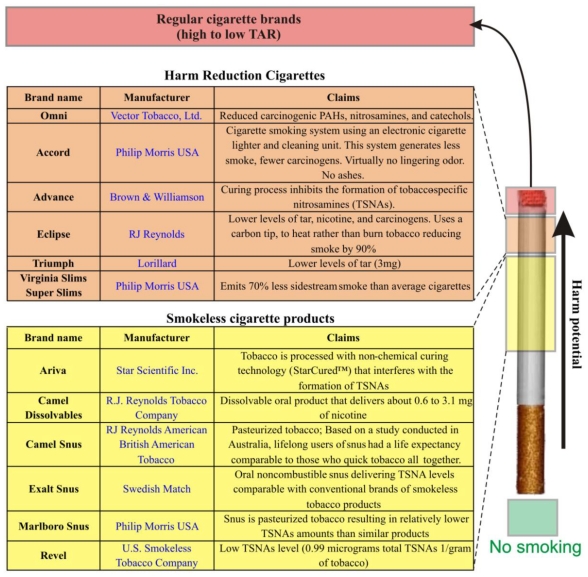
Cigarette products and harm potential. General overview of currently available “harm reduction” and smokeless cigarette products.

Oral nicotine and non-combusted reduced exposure equivalents seem to have higher potential to reduce harm than cigarettes manufactured by altering tobacco and tobacco curing processes because of the lack of combustion byproducts (Figure 3). However, their impact on public health is highly dependent on marketing and public acceptance. Furthermore, the level of exposure to potentially harmful substances including many acknowledged carcinogens is still elevated. In this midst of uncertainties, further studies are also needed to assess how the relative importance of BBB and CNS effects might have shifted with these alternative tobacco products.

## 8. Conclusions

In summary, while the harmful effects of smoking on public health have been well demonstrated the underlying mechanisms of toxicity are not fully understood. At the cerebrovascular level and specifically at the BBB cigarette smoking can severely impair endothelial physiology by directly affecting endothelial tight junctions [56,58] and the ionic homeostasis across the endothelium [57]. The exposure to highly reactive oxygen species generated by cigarette combustion can cause oxidative damage [6,11] and trigger a strong inflammatory cascade that can lead to the onset and/or facilitate the progression of many CNS disorders [45,84,85,96–98].

To date, the scientific evidence is insufficient to evaluate whether PREPs reduce the users’ risk for tobacco-related diseases. Additional independent studies aimed at assessing the potential toxicity of these new products are necessary.
